# Plasmodium vivax Mimicking Morphologic Features of Plasmodium falciparum

**DOI:** 10.7759/cureus.11406

**Published:** 2020-11-09

**Authors:** Najia Ghanchi, Adnan A Khan, Ahmed Raheem, Mohammad A Beg

**Affiliations:** 1 Pathology, Aga Khan University Hospital, Karachi, PAK; 2 Anesthesiology, Aga Khan University Hospital, Karachi, PAK; 3 Emergency Medicine, Aga Khan University Hospital, Karachi, PAK

**Keywords:** plasmodium vivax, mimicking, p.falciparum, throphozoite

## Abstract

Plasmodium vivax (P. vivax) is the most common cause of malaria in Pakistan. Several cases of severe malaria due to P.vivax have been reported from Pakistan and India, however morphological characteristics of the parasite have been mainly ignored. We present two cases of P. vivax mono-infection, which were characterized by multiple infected red blood cells, similar to that seen in Plasmodium falciparum, as observed under microscopy. Both cases were confirmed as mono-infection of P.vivax on Giemsa stained thick and thin films, malaria rapid diagnostic test (RDT) and Polymerase Chain Reaction (PCR). Morphology on peripheral blood smear remains the gold standard for diagnosis of malaria and mimicking morphological features leads to misdiagnosis and mismanagement of patients.

## Introduction

Plasmodium vivax (P. vivax) is the most common cause of malaria in Pakistan and contributes significantly to worldwide morbidity and mortality. An estimated 2.5 billion people are currently at the risk of P. vivax transmission, with approximately 91% of this population located in central and Southeast Asia [1]. In Pakistan, malaria remains an endemic disease with an estimated health care burden of 1.6 million cases annually, of which, 64% of cases can be attributed to P. vivax [2]. 

Although typically regarded as benign, P. vivax has the potential to cause severe disease. In such cases, the clinical profile often mimics that of P. falciparum malaria with renal failure, cerebral malaria and hematologic abnormalities as prominent features [3]. Apart from clinical features, morphological features of P. vivax can also resemble that of P. falciparum. Morphology remains the gold standard for diagnosis of malaria [4]. This may lead to misdiagnosis followed by mismanagement, especially in resources limited areas where other diagnostic modalities like rapid diagnostic test (RDT) and Polymerase Chain Reaction (PCR) are unavailable. Herein, we present two cases where P. vivax was seen to imitate Plasmodium falciparum both clinically and morphologically.

## Case presentation

Case 1

A 57-year-old male presented to the Emergency Department of the Aga Khan University Hospital (AKUH) with a one-day history of fever. The fever was intermittent, associated with chills, rigours, cough, vomiting, watery diarrhoea and progressive drowsiness. His vitals included a temperature of 38.5-degree celsius, tachycardia with a pulse of 105/minute, blood pressure 90/45 mmHg, tachypnea with respiratory rate 25/minute and oxygen saturation was maintained at 98%. On examination, the patient was drowsy but oriented in time, place. He had conjunctival pallor and appeared dehydrated. Respiratory examination revealed bilateral basal crepitation’s. Cardiovascular and abdominal examinations were within normal limits. The neurological assessment revealed a Glasgow Coma Score (GCS) score of 13/15. His pupils were reactive to light but anisocoric (Left pupil 3mm, Right pupil 2mm). Motor examination showed right up to going plantar.

The patient’s arterial blood gases revealed a normal pH of 7.41 but decreased levels of CO_2_ (20.30mmHg), O_2_ (73.80 mmHg) and bicarbonate (12.5 mmHg). Low values of Hb 8.2, white cell count (WCC) normal and low platelets 110 with treatment these became within normal limits. Results of CBC showed low Hb and low platelets as above, and peripheral film, thick and thin film morphology should be added Thick and Thin blood films showed trophozoites and gametocytes of P.vivax, in addition to immuno-chromatography test (ICT) and PCR confirmation of mono-infection with P. vivax. Serum lactate was raised at 7.3 mmol/L.

Coagulation profile revealed an elevated prothrombin time (PT) of 35.4 seconds, a raised INR of 3.42 and an elevated Activated Partial Thromboplastin Time (APTT) of more than 120 seconds. Liver function tests revealed total, direct and indirect bilirubin 2.8 mg/dL, 2.4 mg/dL and 0.4 mg/dL respectively. Liver enzymes were within normal. Dengue antigen was negative. Malaria ICT test was also positive for P.vivax. Radiological studies showed normal chest X-ray and CT scan of the head while an ultrasound examination of the liver revealed a fatty liver. A provisional diagnosis of complicated malaria and acute kidney injury was made. A presumptive diagnosis of acute kidney injury was made on the abnormal Creatinine and electrolytes observed; these became within normal limits with treatment.

He was started on a broad-spectrum antibiotic (Piperacillin and Tazobactam 4.5 mg), and Artemisinin-based combination therapy (Artemether and Lumefantrine), an antiemetic (Metoclopramide 10 mg), and an antipyretic (Paracetamol 1000 mg). Rehydration with a normal saline infusion was started, and the patient’s serum potassium ion levels were replenished. Subsequently, the patient remained vitally stable. He reported a single fever spike at 38degrees Celsius on the second day of admission followed by an uneventful hospital stay. The patient’s platelet levels rose steadily to normal levels, and he was discharged on the 4th day of admission.

A similar case of infection with P. vivax is presented below where P. vivax was seen to parasite P. falciparum in morphology as observed under the microscope. However, no atypical features of disease were observed in this patient. Both cases were confirmed mono-infection of P. vivax on PCR as well.

Case 2

A seven-year-old female, known case of dilated cardiomyopathy, was admitted to the AKUH with a three-day history of fever and increased frequency of urination. The fever was associated with rigours and chills. Her past medical history was significant for a similar episode of fever two weeks prior, which resolved with a course of oral Amoxicillin and Clavulanic acid. Patient vitals at presentation were the temperature of 38-degree Celsius, tachypnea with a respiratory rate of 30 breaths/min, hypotensive with blood pressure 79/38 mmHg, tachypnea with a heart rate of 121 beats/min and decreased oxygen saturation of 91%.

On examination, the patient appeared lean for age (18 kg) but alert and oriented in time and place. She had conjunctival pallor. Cardiovascular, respiratory and abdominal examinations were within normal limits. The neurological assessment revealed a GCS score of 15/15. The rest of the examination was unremarkable.

During the hospital stay, the patient remained febrile and developed thrombocytopenia. Based on clinical symptoms and positive malaria parasite on Giemsa stained blood smears and ICT test, a diagnosis of P. vivax malaria was made. The patient was administered IV Ceftriaxone and Artemisinin-based combination therapy (Artemether and Lumefantrine). The patients’ platelet count rose to normal levels, and she was discharged.

**Figure 1:**
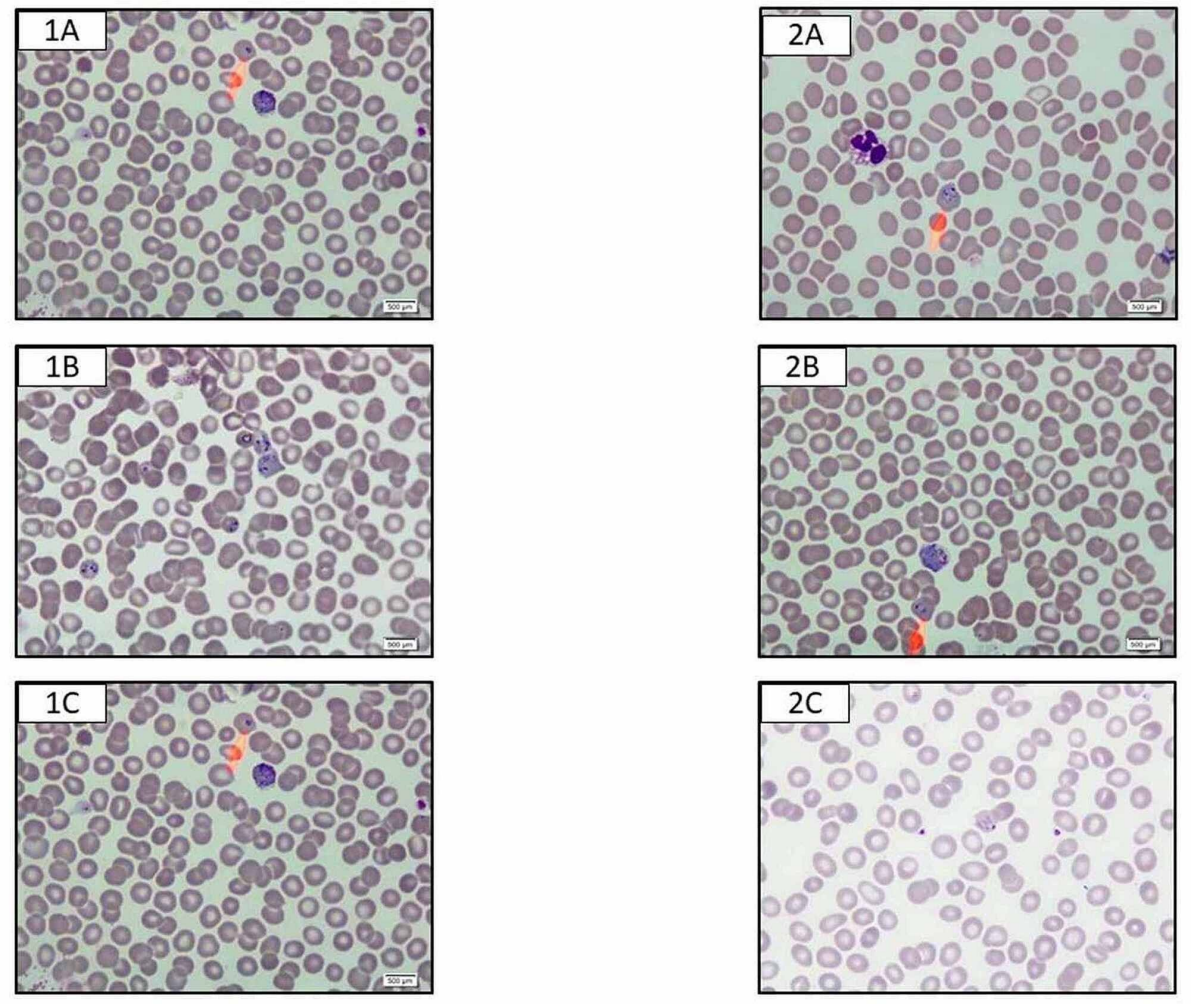
Morphological features of the giemsa stained slides from two cases 1(A-C) and 2 (A-C) which were similar to P.falciparum morphologically showing multiple infected RBCs Plasmodium Falciparum ( P.falciparum), Red blood cells (RBCs)

Morphological features of the Giemsa stained slides from two cases 1 (A-C) and 2 (A-C) which were similar to P. falciparum morphologically showing multiple infected RBCs as presented in figure 1.

## Discussion

P. vivax malaria is commonly associated with chills, vomiting, malaise, headache, fever and myalgia. Recently, however, P. vivax infection has been documented to present as severe malaria previously attributed solely to P. falciparum [5]. The spectrum of clinical manifestations exhibited by P.vivax malaria suggests an interplay between host, parasite and external factors [6-7]. Among these, parasite virulence appears to play an evolving role. Anti-malarial drug resistance, invasion of Duffy negative erythrocytes, production of anti-erythrocyte antibodies, and P. vivax polymorphisms altering cytokine production emphasize the evolution of parasite virulence [8-11]. These observations beg the question of whether morphological similarities exist between P. falciparum and ‘malignant’ P. vivax strains, in addition to similarities in pathophysiology and clinical manifestations. Furthermore, more studies are needed to ascertain whether P. vivax strains causing severe malaria differ morphologically from those causing ‘benign’ disease.

Peripheral blood smear examination is the gold standard for the diagnosis of malaria. For that purpose, we use thick and thin Giemsa-stained blood smears to identify the malarial parasites. Schizonts, trophozoites and gametocytes are commonly seen stages of the malarial parasite; while, cases with an atypical morphology of P. vivax have also been observed.

Recent studies have also suggested similarity in morphology between the Plasmodium species themselves. An imported case from Gabon has revealed up to 6 amoeboid trophozoites in a single red blood cell as well as morphologically abnormal intra-erythrocytic stages of P. vivax that resemble P. ovale [12]. Overlapping morphological features between P. vivax and P. ovale have also been reported from Spain [13]. Furthermore, one study reports atypical falciparum malaria imported from Africa, whose blood smear contained many large trophozoites, with punctiform or massive brown pigment granules; the body shape of this plasmodium specimen was similar to that of P. vivax and P. ovale [14].

Recognition of such atypical morphology of P. vivax is important to avoid diagnostic confusion with spirochetes, trypanosomes and other plasmodia species and to guide effective management and treatment of patients.

## Conclusions

This study can serve as a basis for future studies discussing atypical morphological features of P. vivax and correlating them to the severity of the disease. Should such a correlation be found, it can be used to distinguish complicated from uncomplicated malaria at an early stage to initiate appropriate early treatment and prevent further complications.
